# Microencapsulated curcumin from *Curcuma longa* modulates diet-induced hypercholesterolemia in Sprague Dawley rats

**DOI:** 10.3389/fnut.2022.1026890

**Published:** 2022-10-06

**Authors:** Humaira Ashraf, Masood Sadiq Butt, Iahtisham-Ul-Haq, Muhammad Nadeem, Rana Muhammad Aadil, Alexandru Vasile Rusu, Monica Trif

**Affiliations:** ^1^Department of Food Science and Technology, Jinnah University for Women, Karachi, Pakistan; ^2^National Institute of Food Science and Technology, University of Agriculture, Faisalabad, Pakistan; ^3^Kauser Abdulla Malik School of Life Sciences, Forman Christian College (A Chartered University), Lahore, Pakistan; ^4^Institute of Human Nutrition and Dietetics, Gulab Devi Educational Complex, Lahore, Pakistan; ^5^Life Science Institute, University of Agricultural Sciences and Veterinary Medicine Cluj-Napoca, Cluj-Napoca, Romania; ^6^Faculty of Animal Science and Biotechnology, University of Agricultural Sciences and Veterinary Medicine Cluj-Napoca, Cluj-Napoca, Romania; ^7^Department of Food Research, Centre for Innovative Process Engineering (Centiv) GmbH, Syke, Germany

**Keywords:** curcumin, atherogenic index, microencapsulation, maltodextrin, gelatin, cholesterol, bioavailability, supercritical fluid extraction

## Abstract

Hypercholesterolemia is one of the major causes of cardiovascular ailments. The study has been conducted on the hypothesis that hypercholesterolemia can be modulated by microencapsulated curcumin due to its enhanced bioavailability. In this context, curcumin obtained from fresh rhizomes of *Curcuma longa* by conventional (CSE) and supercritical fluid (SFE) extractions, has been successfully microencapsulated using a mixture of gelatin and maltodextrin. The microencapsulated curcumin _CSE_ _&SFE_, has been added as supplemented diet and has been resulted in maximum plasma concentration of curcumin at 100 min as 529.31 ± 8.73 and 405.23 ± 7.12 μg/mL, respectively compared to non-encapsulated turmeric powder used as control. During the bio evaluation trial, turmeric powder (3%), microencapsulated curcumin_CSE_ (1%) and microencapsulated curcumin_SFE_ (0.5%) were provided to designate rat groups categorized by normal; N_1_, N_2_, and N_3_ and hypercholesterolemic; H_1_, H_2_, and H_3_ conditions, respectively. The incorporation of microencapsulated curcumin_SFE_ in the supplemented diet caused a reduction in serum cholesterol, low density lipoprotein (LDL) and triglycerides, athrogenic index (AI) and cardiac risk ration (CRR) as 5.42 and 12.81%, 7.25 and 15.42%, 3.17 and 9.38%, 15.38 and 29.28%, and 10.98 19.38% in normo- and hypercholesterolemic rat groups. Additionally, high-density lipoprotein (HDL) and anti-atherogenic index (AAI) indicated a significant increase in all treated rat groups. Conclusively, the inclusion of turmeric and curcumin microencapsulates in the dietary module has been proven effective to alleviate hyperlipidemia. Therefore, the present study is proven that curcumin absorption *via* the gastrointestinal tract and its stability toward metabolization in the body increased *via* microencapsulation using maltodextrin and gelatin. Microencapsulated curcumin reaches the target site *via* oral administration because of sufficient gastrointestinal residence period and stability in the digestive tract.

## Introduction

Globally, the incidence of cardiovascular diseases (CVDs) is escalating owing to poor living patterns and unwise food choices. Furthermore, hyperlipidemia is amongst the leading causes of CVDs, characterized by increased cholesterol and triglyceride levels. Cholesterol is one of the lipophilic compounds that is circulated by chylomicron, low-density lipoproteins (LDL), very low-density lipoproteins (vLDL) and HDL as carriers (1, 2). A progression of LDL oxidation, which is lethal to endothelial cells, can be caused by poor eating habits over an extended period. In this way the oxidative balance of the body is disturbed, further resulting in plaque formation in blood vessels by oxidizing lipoproteins (3).

Plants have always been a part of traditional health treatments since antiquity around the globe (4, 5). Isolated bioactive molecules from spices may serve as preliminary materials for diet-based therapies. Amongst, curcumin is the principal turmeric bioactive moiety and accounts for 75–80% of total curcuminoids. Curcuminoids are secondary plant constituents that are extracted from the rhizome of various Curcuma species. It is aryl-C7-aryl, exhibiting a diarylheptanoid structure responsible for the orange-red shade of turmeric (6, 7). Unfortunately, the bioavailability of curcumin is a challenge as a merely insignificant fraction of curcumin reaches the target site *via* oral administration because of insufficient gastrointestinal residence period, low absorption through the intestinal wall and instability in the acidic environment of digestive tract (8). The reseason behind its limited stability is rapid convertion into ferulic acid and vanillin by intestinal enzymes and conjugation with glucuronide and sulfate in hepatic cells, thus lowering systemic concentration (9, 10). To extract the maximum therapeutic outputs from curcumin, it is necessary to enhance the bioavailability of this bioactive compound. Purposely, various techniques have been tested to enhance the bioavailability of curcumin with improved food applications. A variety of methods are available to increase the bioavailability, e.g., the addition of piperine, micellation of curcuminoids or nanotechnological methods. Among all of these, curcumin encapsulation at micro-level using lyophilization is gaining more popularity due to its suitability for heat sensitive biomolecules and versatility for utilizing various coating materials composed of sugars, proteins, lipids, gums, native and modified polysaccharides and synthetic polymers whilst considering the compatibility with the core ingredient i.e., curcumin. In this context, foregoing explorations on coating of micro/nano-food particles with compatible material have assured high encapsulating efficiency and shelf life of bioactive compounds. Furthermore, the absorption and stability of curcumin against metabolization *via* gastrointestinal tract may be improved (11).

Numerous epidemiological studies provide convincing evidence regarding the use of curcumin to prevent coronary diseases (8, 12). Earlier literature revealed that curcumin blocks the aggregation of platelets and improves erythrocyte fragility, which are important factors in the pathogenesis of heart attack and arteriosclerosis. Moreover, the administration of curcumin through a daily diet tends to enhance the activity of hepatic acyl-CoA that hinders excessive fat accumulation in adipose tissues and the liver (13). Turmeric possesses hepatoprotective properties due to its cholesterol-lowering effects. It was confirmed by research outcomes of Elahi (14) on rat models concluding that curcumin intakes decreases the absorption of saturated fat by rapidly eliminating bile hence lowering the risk of heart diseases.

The mechanistic approach related to curcumin revealed the regulatory effect on the activity of hepatic cholesterol 7-α-hydroxylase thus normalizing the biosynthesis of bile acids. Since no drug is deprived of any side effects, it is therefore recommended to attenuate hypercholesterolemia using diet-based therapies having no or low toxic effects as compared to pharmaceuticals. It is interesting to know that curcumin down-regulates gene expression for 3-hydroxy-3-methyl-glutaryl-co-enzyme A reductase (HMGR) through transcriptional inhibition. The major objective of cholesterol management is to attenuate serum LDL levels (15). The proposed mechanism described that curcumin decreases the level of Apo-B lipoprotein in serum from hepatocytes. Apo-B lipoprotein is involved in LDL transfer to extrahepatic cells at a 1:1 ratio (one Apo-B lipoprotein can bind one LDL). Moreover, regulating HDL to manage hyperlipidemia is considered good as it sets back the transport of cholesterol to the liver for the removal of triglycerides (TG) and cholesterol bodies along with bile acid (16). Unfortunately, there is a lack of sufficient data regarding the use of microencapsulated curcumin isolated using a supercritical fluid extraction system and conventional solvent, i.e., ethanol in comparison with turmeric to address dyslipidemia. In this study, it has been expected that due to microencapsulated curcumin, which has a higher bioavailability than turmeric, it could be possible to reduce diet-induced hypercholesterolemia. As a result, the current research was designed to investigate the hypocholesterolemic potential of turmeric and microencapsulated curcumin obtained from dried rhizomes of *Curcuma longa* by conventional (CSE) and supercritical fluid extracts (SFE) using male rats in a diet module.

## Materials and methods

Fresh rhizomes of turmeric (Kasur) were procured from Ayub Agriculture Research Institute, Faisalabad, Pakistan. Analytical and HPLC grade reagents and standards were purchased from Merck (Merck KGaA, Darmstadt, Germany) and Sigma-Aldrich (Sigma-Aldrich Tokyo, Japan). For an efficacy study, Male Sprague Dawley rats were acquired and housed in the Animal Room of NIFSAT. For biological assays, diagnostic kits were purchased from Sigma-Aldrich, Bioassay (Bioassays Chemical Co. Germany) and Cayman Chemicals (Cayman Europe, Estonia).

### Extraction of curcumin

Fresh rhizomes of *Curcuma longa* (turmeric variety: Kasur) were purchased from Ayub Agriculture Research Institute (AARI), Faisalabad, Pakistan. The turmeric was then cleaned, and adherent soil or other matter was separated followed by dehydrating it at 60°C in a lab-scale dehydrator. The dried turmeric was ground to a fine powder and kept in an airtight glass container till further utilization. Curcumin from dehydrated turmeric powder was isolated using two different protocols. Firstly, aqueous ethanol (50% v/v) was used to extract curcumin from turmeric powder by agitation at a constant temperature of 50°C for 65 min following the prescribed procedures of Kulkarni et al. (17). Afterward, Rotary Evaporator (Eyela, Japan) was used to concentrate the filtered ensuing curcumin extract and termed conventional solvent extract (curcumin_CSE_) containing 31.48 ± 1.35 mg/g of curcumin in it. Secondly, SFE of dried turmeric powder was obtained using CO_2_ as supercritical fluid through supercritical fluid extraction (SFT-150) system. The sample was placed in an extraction vessel and CO_2_ was introduced to the vessel and a stay time of 150 min was given (18). After completion of the cycle, the extracted curcumin is recovered in a glass vial. This curcumin extract was encoded as SFE (curcumin_SFE_) containing 52.41 ± 2.38 mg/g of curcumin in it (19).

### Microencapsulation of curcumin

Curcumin was encapsulated using homogenous emulsions comprised of maltodextrin (20 g) and gelatin (6 g) per 100 g solution. For this purpose, gelatin was dissolved in warm distilled water and mixed with maltodextrin solution. Afterward, turmeric extracts were added at a concentration of 10% depending on the weight of encapsulating material. The mixture was homogenized for 10 min at 3,500 rpm. The prepared emulsions were kept at –35°C for 24 h following lyophilization at –30°C according to the prescribed method of Malacrida and Telis (11). The resultant material was finely ground and stored for evaluation trial.

### Bioavailability of curcumin

The study was approved by the Directorate of Graduate Studies, UAF (No. DGS/474-79) after ensuring the standards for handling and care of laboratory animals from the Departmental Bioethics Committee. To access the bioavailability of encapsulated curcumin, already acclimatized (23 ± 2°C temperature, 55 ± 5% relative humidity and 12 h light/dark cycle for 1 week before study) male Sprague Dawley rats (*n* = 10 per group; power analysis) were randomly distributed into three groups. The turmeric powder (3%) enriched diet was orally administrated to the G_0_ group whereas, G_1_ and G_2_ were provided with microencapsulated curcumin_CSE_ (1%) and microencapsulated curcumin_SFE_ (0.5%) enriched diets, respectively. To quantify the curcumin content in plasma, samples were collected at time intervals of 50, 100, 150, and 200 min (20).

### Bioevaluation trial for a hypolipidemic effect

Male Sprague Dawley rats were used and National Research Council’s Guide for the Care and Use of Laboratory Animals were followed during the entire study. The efficacy trial was performed to test the therapeutic potential of curcumin-supplemented diets against hypercholesterolemia. The rats were acclimatized for a week by feeding on a control diet alongside controlling temperature (23 ± 2°C) and relative humidity (55 ± 5%) and then randomly assigned to different groups as illustrated in Table 1.

**TABLE 1:** Bioefficacy plan.

Groups	Description
N_0_	Normal rats reared on a normal diet
N_1_	Normal rats reared on a turmeric powder containing diet
N_2_	Normal rats reared on Microencapsulated curcumin_CSE_ containing diet
N_3_	Normal rats reared on Microencapsulated curcumin_SFE_ containing diet
H_0_	Hypercholesterolemic rats reared on a normal diet
H_1_	Hypercholesterolemic rats reared on a turmeric powder containing diet
H_2_	Hypercholesterolemic rats reared on Microencapsulated curcumin_CSE_ containing diet
H_3_	Hypercholesterolemic rats reared on Microencapsulated curcumin_SFE_ containing diet

CSE, Conventional solvent extract; SFE, Supercritical fluid extract.

A Bioevaluation trial (60 days) was conducted on normal and hypercholesterolemic rats where hypercholesterolemia was induced by administrating a high cholesterol diet to the rats. Each rat group (*n* = 10) was given the respective dietary module during the whole trial (Table 2). In this connection, N_1_ and H_1_ were reared on turmeric powder @ 3% enriched diet, whilst N_2_ and H_2_ were provided with microencapsulated curcumin_CSE_ @ 1% enriched diet. The rats in N_3_ and H_3_ were fed on a 0.5% microencapsulated curcumin_SFE_ enriched diet whereas, N_0_ (negative control) and H_0_ (positive control) were used as reference controls. At the end of the trial, blood samples of overnight fasted rats were collected and centrifuged (4,000 rpm for 6 min) for collection of sera *via* centrifuge machine. The collected sera were subjected to assessment of various lipidemic biomarkers using Microlab 300, Merck, Germany. Furthermore, lipidemic ratios were also calculated to assess the risk index for cardiac diseases.

**TABLE 2:** Diet composition for different rat groups.

Ingredients	Quantities (g/1,000 g)							
	N_0_	N_1_	N_2_	N_3_	H_0_	H_1_	H_2_	H_3_
Flour	812	782	802	807	769.6	739.6	759.6	764.6
Corn oil	90	90	90	90	120	120	120	120
Casein	50	50	50	50	50	50	50	50
Minerals	30	30	30	30	30	30	30	30
Vitamin	10	10	10	10	10	10	10	10
Bran	8	8	8	8	5	5	5	5
Cholesterol	–	–	–	–	15	15	15	15
Choline	–	–	–	–	0.4	0.4	0.4	0.4
Turmeric powder	–	30	–	–	–	30	–	–
Microencapsulated curcumin_CSE_	–	–	10	–	–	–	10	–
Microencapsulated curcumin_SFE_	–	–	–	5	–	–	–	5

### Serum lipid profile

Serum lipid profiles including total cholesterol (TC), LDL, HDL, and TG were measured following respective protocols. Sera cholesterol and LDL were estimated by CHOD–PAP method and TG were estimated by GPO–PAP method as described by Kim et al. (21). HDL in sera was measured by the Cholesterol Precipitant procedure as described by Alshatwi et al. (22). However, VLDL and non-HDL levels in sera were calculated using Friedewald expression. Moreover, Atherogenic Index (AI), HDL to TC ratio (HTR%), Cardiac Risk Ratio (CRR) and Anti-Atherogenic Index (AAI) were computed using the expressions mentioned by Ashfaq et al. (23).

### Statistical analyses

One-way Analysis of Variance (ANOVA) was used to determine the level of significance using Cohort version 6.1. The *P*-value < 0.05 was considered a significant effects. Furthermore, Tukey’s Honest Significant Difference (HSD) test was used for *post hoc* comparison of the means. All the results are then expressed as mean ± SD.

## Results

### Bioavailability of curcumin

**FIGURE 1:**
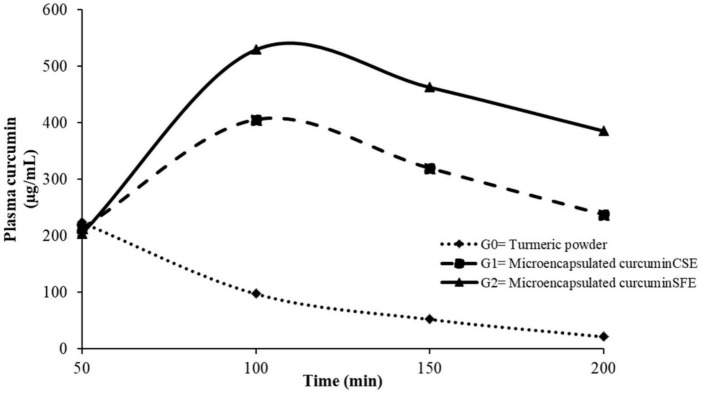
Curcumin concentration (μg/mL) in rat plasma at different time intervals.

Data obtained for curcumin bioavailability in plasma samples of rats were statistically analyzed. Due to the influence of treatments and time intervals, there was a considerable fluctuation in curcumin content in rat plasma (Figure 1). The maximum value (C_max_) for curcumin (529.31 ± 8.73 μg/mL) was observed for the rat group (G_2_) that was provided with a microencapsulated curcumin_SFE_ enriched diet with a maximum time (T_max_) of 100 min that decreases to 462.98 ± 7.25 and 385.76 ± 5.01 μg/mL at 150 and 200 min, respectively (Figure 1). For the G_1_ group rats fed on a microencapsulated curcumin_CSE_ enriched diet, the recorded values for curcumin in rat’s plasma were 223.51 ± 3.76, 405.23 ± 7.12, 319.57 ± 6.41, and 237.49 ± 4.25 μg/mL at 50, 100, 150, and 200 min, respectively. However, curcumin concentration decreased rapidly from 205.45 ± 3.84–21.29 ± 0.14 μg/mL in plasma of rat group (G_0_) fed on a diet containing turmeric powder from 50 to 200 min, respectively. The trends observed in these results showed that the bioavailability of curcumin is enhanced using microencapsulation which is one of the limiting factors in the effectiveness of curcumin’s medicinal worth.

### Cholesterol

The data for the serum lipid profile of male Sprague Dawley rats after a 60-day feed model trial is depicted in Table 3. The statistical analysis of the pooled data regarding TC in normal and high cholesterol diet-induced hypercholesterolemic rat groups depicted significant differences (*P* < 0.05) among treated groups for 2 months of study. It was observed that in the normal group TC decreased in rat groups fed on a diet containing turmeric powder, microencapsulated curcumin_CSE_ and microencapsulated curcumin_SFE_, respectively as compared to rats treated with a normal diet. On the other side, the TC of rats (H_0_) fed with a diet containing high cholesterol increased by 43.72% in contrast to its normal counterpart (N_0_). These outcomes showed that the cholesterol administration in the daily diet caused lipid metabolic dysfunction, ultimately endorsing hyperlipidemia. However, variations in dietary patterns of rat groups by the inclusion of turmeric powder, curcumin_CSE_ and curcumin_SFE_ microencapsulated alleviated total sera cholesterol as signified in H_1_ (7.32%), H_2_ (10.54%) and H_3_ (12.81%) groups, respectively. The current findings show that supplementing the diets with curcumin microencapsulated lowers circulating lipids, alleviating the negative effects of treated rats’ hypercholesterolemia.

**TABLE 3:** Serum lipid profile of rats treated with different curcumin-enriched diets.

Groups	High density lipoproteins (mg/dL)	Low density lipoproteins (mg/dL)	Triglycerides (mg/dL)	Total cholesterol (mg/dL)	Very low density lipoprotein (mg/dL)	Non-high density lipoprotein (mg/dL)
N_0_	34.87 ± 1.25^c^	31.73 ± 1.30^d^	64.31 ± 2.18^d^	80.22 ± 2.72^d^	12.86 ± 0.23^c^	45.35 ± 1.76^d^
N_1_	35.42 ± 1.31^c^	30.98 ± 1.16^de^	63.08 ± 2.45^d^	78.34 ± 2.89^d^	12.61 ± 0.45^c^	42.92 ± 0.97^de^
N_2_	35.89 ± 1.56^c^	29.77 ± 1.03^e^	62.59 ± 2.39^de^	76.85 ± 3.18^de^	12.51 ± 0.19^c^	40.96 ± 1.41^e^
N_3_	36.01 ± 1.72^c^	29.23 ± 0.99^d^	62.27 ± 1.92^e^	75.87 ± 3.41^e^	12.45 ± 0.57^c^	39.86 ± 2.12^e^
H_0_	59.30 ± 2.07^b^	58.15 ± 2.26^a^	97.02 ± 4.36^a^	142.56 ± 6.42^a^	19.40 ± 0.71^a^	83.26 ± 2.78^a^
H_1_	60.55 ± 2.24^ab^	52.41 ± 1.61^b^	91.69 ± 3.32^b^	130.12 ± 4.70^b^	18.33 ± 0.62^ab^	69.57 ± 3.09^b^
H_2_	61.48 ± 2.26^a^	50.14 ± 2.06^bc^	89.53 ± 3.60^bc^	127.53 ± 4.97^bc^	17.90 ± 0.49^b^	66.05 ± 1.82^bc^
H_3_	62.31 ± 2.04^a^	49.18 ± 1.87b^c^	87.91 ± 3.52^c^	124.29 ± 5.09^c^	17.58 ± 0.33^b^	61.98 ± 1.95^c^

Data values represent mean ± SD (*n* = 10); means carrying different superscripted letters in a column differ significantly (*P* < 0.05).

### High-density lipoprotein

The means relating to HDL expounded significant differences (*P* < 0.05) for normal as well as hyper cholesterolemic rat groups (Table 3). The diet-induced hypercholesterolemic rat group reared on a regular diet (H_0_) illustrated a marked reduction in HDL level as compared to its negative control group (N_0_) illuminated the highly damaging impact of high cholesterol diet on lipidemic biomarkers, HDL. Nonetheless, it is unusual to mention that a diet containing turmeric powder (H_1_), nutraceutical microencapsulated-curcumin_CSE_ (H_2_) and –curcumin_SFE_ (H_3_) restored the HDL cholesterol more significantly than H_0_. However, a comparative study for normal and hypercholesterolemic rats showed that differences in sera HDL cholesterol were statistically similar in normal rats although marginally better HDL levels were recorded in N_1_, N_2_, and N_3_ rats treated with relevant turmeric powder and curcumin microencapsulated than N_0_ indicating a positive effect of turmeric polyphenol curcumin that helps to maintain the needed level of good cholesterol, which prevents plaque development in the body’s systemic blood circulation.

### Low-density lipoprotein

The statistical data regarding LDL cholesterol in normal rats depicted a higher level of LDL in sera of control rats (N_0_) than turmeric powder, nutraceutical microencapsulated-curcumin_SFE_ (N_2_) and –curcumin_CSE_ (N_1_) treated rats. Furthermore, remarkable differences (*P* < 0.05) were recorded related to LDL cholesterol among the groups of hyperlipidemic rat models. The highest LDL value was noted in the positive control group (H_0_) of hypercholesterolemic rats. The cholesterol feeding throughout the experimental period disturbed lipid metabolism in rats thus elevating serum LDL cholesterol. Nonetheless, the provision of curcumin-based diet to hypercholesterolemic rats was proven effective to manage lipid-related anomalies by suppressing the elevated LDL level as illustrated by their mean values relating to H_1_, H_2_, and H_3_. Compared to H_0_, a significant 15.42, 13.77, and 9.86% reduction in LDL levels of H_1_, H_2_ and H_3_ groups correspondingly were observed validating the therapeutic potential of a turmeric polyphenol enriched diet. In a nutshell, diet containing a variety of turmeric bioactives could be beneficial in reducing a variety of lifestyle-related discrepancies associated with serum lipid abnormalities.

### Very low-density lipoprotein

From the results, it is obvious that rats depending on a high cholesterol diet in group H_0_ got higher (33.71%) VLDL cholesterol than that of N_0_ thus, demonstrating the adverse impact of high cholesterol on balanced lipidemic parameters as about 50% of a VLDL particle is composed of TG. N_1_, N_2_ and N_3_ were statistically alike when compared to N_0_, whereas, significant variations (*P* < 0.05) were specified compared to H_0_, H_1_, H_2_, and H_3_. However, in terms of VLDL, when compared to H_0_, supplementation of turmeric powder, nutraceutical curcumin isolated using conventional solvent and supercritical fluid in the dietary module of H_1_, H_2_, and H_3_ rats’ groups managed to lower VLDL cholesterol by 5.52, 7.73, and 9.38%, correspondingly highlighting protective effects of curcumin-based diet (Table 3).

### Non-high-density lipoprotein

The data for normal and diet-induced hypercholesterolemic rat groups (Table 3) elucidated the significant efficacy of the study of microencapsulated curcumin in reducing serum non-High-density lipoprotein (n-HDL) levels for all treated groups. Administration of turmeric powder (H_1_), microencapsulated-curcumin_CSE_ (H_2_) and –curcumin_SFE_ (H_3_) showed a significant impact on n-HDL indicating modulatory effect of turmeric bioactive compound, i.e., curcumin on the major lipoproteins linked with a higher risk of cardiovascular disease. When compared to H_0_, diet modification by using turmeric for H_1_, H_2_, and H_3_ lowered n-HDL by 16.44, 20.67, and 25.56%, correspondingly. Similarly, the normal counterparts of H_1_, H_2_, and H_3_, that is, N_1_, N_2_, and N_3_ groups also presented significantly lower n-HDL levels.

### Triglycerides

Triacylglycerols are a major element of chylomicron and may act as energy substrates for hepatic and peripheral tissues, chiefly, muscles. The pooled means (Table 3) revealed an obvious trend in which TG in the serum of three test groups (N_1_, N_2_, and N_3_) was lower than the rat group fed a normal diet (N_0_). However, triglyceride level of hyperlipidemic rat groups reared on diet containing turmeric powder (H_1_), microencapsulated-curcumin_CSE_ (H_2_) and –curcumin_SFE_ (H_3_) was dramatically lower than that of H_0_ (*P* < 0.05). The momentous decline was observed in H_3_ (9.38%) followed by H_2_ (7.72%) and H_1_ (5.49%), respectively as referred from H_0_. It indicated that Curcumin-rich diets reduce the negative effects of high cholesterol consumption on lipid profiles.

### Lipidemic ratios

In terms of lipidemic ratios, the rats in the H_0_ group had a significantly higher AI (*P* < 0.05) than rats in the N_0_ group due to high cholesterol in their diet. There was a significant difference between H0 and the other study groups (Table 4). The variations in this parameter among groups were attributed to the provision of curcumin-enriched diets that normalized the atherogenic index; a novel indicator involved in dyslipidemia by normalizing HDL levels. It caused an 18.57, 23.57, and 29.28% reduction in the AI in H_1_, H_2_, and H_3_, respectively than H_0_. The alike trend was observed for other study groups relying on a normal diet. In contradiction, the AAI of all rat groups increased momentously as compared to their control groups N_0_ and H_0_ due to the positive effect of curcumin on good cholesterol (HDL) in sera. The risk of cardiac arrest due to plaque formation in arteries is increased by elevation in LDL serum cholesterol, hence increasing the CRR.

**TABLE 4:** Lipidemic ratio of rats treated with different curcumin-enriched diets.

Groups	AI	Cardiac risk ratio (CRR)	HDL:TC ratio (HTR%)	AAI
N_0_	1.30 ± 0.02^b^	0.91 ± 0.03^b^	43.47 ± 1.72^bc^	0.77 ± 0.01^bc^
N_1_	1.21 ± 0.01^bc^	0.87 ± 0.02^bc^	45.21 ± 1.37^b^	0.83 ± 0.04^b^
N_2_	1.14 ± 0.05^b^	0.83 ± 0.01^c^	46.70 ± 0.79^b^	0.88 ± 0.01^c^
N_3_	1.10 ± 0.02^bc^	0.81 ± 0.01^cd^	47.46 ± 2.45^ab^	0.90 ± 0.03^ab^
H_0_	1.40 ± 0.03^a^	0.98 ± 0.07^a^	41.60 ± 1.98^c^	0.71 ± 0.02^c^
H_1_	1.14 ± 0.01^b^	0.87 ± 0.04^bc^	46.53 ± 2.13^b^	0.87 ± 0.04b^c^
H_2_	1.07 ± 0.06^bc^	0.82 ± 0.02^c^	48.20 ± 1.56^ab^	0.93 ± 0.06^b^
H_3_	0.99 ± 0.02^c^	0.79 ± 0.05^d^	50.13 ± 0.66^a^	1.01 ± 0.05^a^

Data values represent mean ± SD (n = 10); means carrying different superscripted letters in a column differ significantly (P < 0.05).

According to statistical inferences for CRR (Table 4), turmeric and curcumin inclusion isolated by conventional ethanol and super-critical fluid in daily intakes of different rat groups had a significant effect on CRR value. The rat groups N_1_, N_2_, and N_3_ are at lower cardiac risk in comparison to N_0_. Likewise, the higher cardiac risk was recorded in H_0_ that was remarkably lower for rat groups feeding on turmeric powder (H_1_) and curcumin_*CSE*_ (H_2_) and curcumin_SFE_ (H_3_) microencapsulated containing diet proving its prophylactic role against elevated lipid biomarkers (LDL, VLDL, and TG). It is also noteworthy, that turmeric and its bioactive; curcumin significantly improves the HTR (%) by upgrading the level of HDL cholesterol in normal (N_1_, N_2_, and N_3_) and high cholesterol (H_1_, H_2_, and H_3_) treated rat groups as compared to negative (N_0_) and positive (H_0_) controls during 2-month efficacy trial.

## Discussion

Nowadays, increased reliance on hypercaloric foods, poor dietary habits and a deskbound lifestyle are the dominant factors throughout the world, ultimately responsible for the escalated prevalence of lifestyle-related disorders such as hyperlipidemia, coronary complications, etc. To cope with this scenario, consumers have been curious about healthy food options to ensure disease prevention beyond basic nutrition. Accordingly, bioactive plant ingredients have captured the attention of health-conscious consumers owing to their acceptability, low cost and safe nature (24). Numerous evidence have enlightened the affirmative participation of spices in improving physiological functionality (25, 26). In this regard, turmeric has gained great attention due to its antioxidant potential, which is primarily attributed to curcumin. Considering the aforementioned facts, the purpose of this study was to evaluate the nutraceutical value of locally cultivated turmeric against high serum cholesterol levels caused by diet (27). To secure these health benefits, it is noteworthy to select optimum extraction modes (conventional and non-conventional techniques), solvent (polar and non-polar), extraction parameters (temperature, pH and time) to isolate curmin from parent plant; turmeric. A study relating to aforementioned parameters was conducted (19). They concluded that green extraction technologies (SFE) should be employed to isolate and purify heat labile food components as compared to conventional extraction tools. Additionally, the low stability and escalated intestinal metabolism of curcumin during first pass metabolism have limited its therapeutic value against various maladies. However, encapsulating the core material in bio-stable matrices improves its bioavailability in systemic circulation. In pharmacokinetic study, Takahashi et al. (28) determined the impact of lecithin liposome as coating material to modulate curcumin release at targeted tissues in rats. Accordingly, 319.2 μg/mL was recorded at 120 min in rat plasma receiving Liposome Encapsulated Curcumin (LEC) as compared to group fed on free curcumin with maximum concentration (C_max_) of 34.6 μg/mL at T_max_ 30 min. It was studied that ingestion of turmeric biomolecule “curcumin” decreases the absorption of high cholesterol diet by rapidly eliminating bile hence lowering the risk of heart diseases (14).

The result of this research supported the modulatory effect of turmeric and microencapsulated curcumin against hyperlipidemia. The outcomes of the research firmly supported the established hypothesis for the research and concluded that microencapsulated curcumin modulates diet-induced hypercholesterolemia due to its higher bioavailability in contrast to turmeric powder. As it was observed that curcumin encapsulated in maltodextrin and gelatin is less susceptible to degradation into metabolites and conjugation. The graphical representation for microencapsulated curcumin availability in rat plasma has confirmed its maximum retention at 100 min as compared to turmeric powder. Another important parameter of the study was green extraction technology, i.e., supercritical fluid extraction used for improved curcumin yield extraction and eco-friendly compared to the conventional extraction method. It is worth noting that the efficacy of biomolecules can be improved by employing novel extraction tools in conjunction with various combinations of bio-stable matrices.

Diet-induced hypercholesterolemia in model feed trials has been used as a routine approach to check the effects of functional compounds against dyslipidemia (23). The results of our study are quite in line with the previous reports. One of the mechanistic approaches behind the hypocholesterolemic potential of turmeric bioactive is to modulate liver X receptor-α, i.e., nuclear receptor protein that regulates macrophage formation and transcriptional factors ultimately maintaining lipid homeostasis (29). It has been reported that the consumption of a high-fat diet (HFD) caused deposition of inflammatory cells, lipid infiltration and localization of Intercellular Adhesion Molecule-1 (ICAM-1) and Vascular Cell Adhesion Molecule-1 (VCAM-1), which are key indicators of coronary heart diseases. However, administration of curcumin did not show any mark of ICAM-1 and VCAM-1 adhesion molecules in the aortic arch of rats fed on high cholesterol diet (16, 30, 31). Later on, Mahmoud et al. (32) investigated the prophylactic effects of curcumin against the hyperlipidemia induced by feeding rats on a HFD. The data proved that curcumin modulates hepatic lipid levels. Accordingly, curcumin may decrease circulatory lipids by hindering adipocytes’ fatty acid synthase (FAS), consequently inhibiting the hepatic lipid accumulation due to suppression in transport of lipid to the liver.

Another theory behind the lipid-lowering capacity of curcumin is the activation of peroxisome proliferator-activated receptor-α (PPAR-α) that accelerates the gene regulating cholesterol transport and fatty acid oxidation thus lowering hepatic cholesterol (33). Curcumin can maintain a balance between β-lipoprotein and α-lipoprotein, which are imperative for the structural and functional integrity of both LDL and HDL, respectively (1).

HDL is necessary to clear TG and cholesterol esters from plasma to be secreted in bile. Curcumin improves HDL levels by reducing the transfer of cholesteryl esters from HDL to LDL (16) thus improving the atherogenic index. In the limelight of another theory, high apolipoprotein A, i.e., precursor of HDL leads to elevated oxido-resistant lipoprotein resultantly, improving the AAI. In this context, curcumin up-regulates the level of apolipoprotein A and acetyltransferase that are involved in cholesterol transport to the liver (34, 35). It is concluded from the aforesaid discussion that turmeric is helpful against cardiovascular complications owing to its positive impact on HDL levels.

Oxidation of LDLs is of prime importance for the progression of arteriosclerosis, which damages the inner lining of endothelial cells. In this connection, curcumin provides cellular integrity and blocks platelet aggregation and peroxidation of lipids thus minimizing the chances of plaque formation in arteries (3, 36). An array of evidence exposed an inverse relation between curcumin consumption and lipid irregularities as it scavenges free radicals and reverses LDL oxidation hindering the formation of foam cells and its deposition in aortic arteries alongside improvement in HDL further coupled with its hypocholesterolemic potential (37). Curcumin directly interacts with hepatic cells for the translation of mRNA that encodes to increase LDL receptors on liver cells. As a result of the increase in LDL receptors, liver cells may sweep up the body’s greater levels of LDL (14). In the current study, the provision of curcumin-based diet to hypercholesterolemic rats was proven to be effective in managing lipid-related abnormalities by suppressing the elevated LDL levels. It can be summarized that turmeric-supplemented foods are useful in alleviating a variety of lifestyle-related ailments.

Hyperlipidemia interrupts the redox balance and leads to irregular and uncontrolled release of free radicals ensuring accelerated atherogenic cases by developing a state of oxidative stress (38). But in the current scenario, the AI was significantly decreased due to the provision of curcumin, which depicts the hypocholesterolemic potential of curcumin. Therefore, a diet containing turmeric, and especially curcumin is valuable to attenuate the risk indices for cardiac ailments by elevating HDL and reducing LDL.

The current findings are consistent with the findings of Chandrakala and Tekulapally (39) who investigated the hypolipidemic effect of turmeric curcumin to alleviate the negative effects of hypercholesterolemia. They reported that both turmeric and curcumin decreased TC, TG, and LDL along with simultaneous increment in HDL concerning control. However, curcumin depicted a more pronounced effect on lipid profile than that turmeric. Another approach elucidated that curcumin upregulates cholesterol-7α-hydroxylase in the hypercholesterolemic rats *via* nuclear receptor liver X receptor (LXR), i.e., responsible for the catabolism of cholesterol in bile acid. Thus, the hypocholesterolemia potential of curcumin is attributed to increased excretion of cholesterol and bile acid from the body *via* feces which reduces cholesterol reabsorption from dietary sources. Moreover, curcumin intakes regulate AMP-dependent kinase and peroxisome proliferator regulated receptors, engaged in the catabolism of adipocytes present in subcutaneous layer (32, 40). Recently, Iwueke et al. (41) have proven that a daily intake of turmeric powder (200 mg/kg) considerably lowers the TC and TG levels in sera. Another meta-analysis conducted on patient with metabolic disorder, Type II diabetes mellitus showed beneficial effect on lipid parameters on relying on diet supplemented with curcumin (42). The previous literature showed that consumption of high fat diet caused deposition of inflammatory cells, lipid infiltration and localization of Intercellular Adhesion Molecule-1 (ICAM-1) and Vascular Cell Adhesion Molecule-1 (VCAM-1) that are key indicators of coronary heart diseases. However, curcumin significantly reduced ICAM-1 and VCAM-1 adhesion molecules in aortic arch of rats fed on high cholesterol diet (16). Another theory behind lipid lowering capacity of curcumin is the activation of PPAR-α (peroxisome proliferator activated receptor-α) that accelerates the gene regulating cholesterol transport and fatty acid oxidation thus lowers the hepatic cholesterol. Although, the benefits of turmeric and its biologically active constituent curcumin for the treatment of hyperlipidemia have been discussed in depth. However, further research on genetic aspects of the modulatory effects and the underlying mechanisms of microencapsulated curcumin are yet to be uncovered so that a successful nutraceutical or dietary regimen can be recommended for humans.

## Conclusion

Microencapsulation helps to incorporate sensitive ingredients into foods, food supplements or pharmaceuticals. This study showed that curcumin absorption *via* the gastrointestinal tract and the stability toward metabolization in the body can be increased *via* microencapsulation using maltodextrin and gelatin. In this way, microencapsulated active ingredients can be released with a delay without catabolizing and being conjugated in first pass, thus increase gastric tolerance compared to conventional forms such as rapidly disintegrating tablets. Furthermore, the current research has provided evidence that turmeric has nutraceutical worth to alleviate hyperlipidemia. A diet containing turmeric or curcumin can modulate lipid profile markers. In this context, microencapsulated curcumin showed a remarkable regulatory impact on total cholesterol, LDL and TG along with improvement in HDL of treated rat groups. It was noticed that turmeric powder, microencapsulated-curcumin*_CSE_* and –curcumin*_SFE_* are more effective and biologically active in delivering the therapeutic effects in the hyperlipidemic state than that in normocholesterolemic conditions. Thereby, it is concluded that the use of microencapsulated curcumin could be a sustainable strategy to alleviate cardiovascular complications *via* dietary therapies. Nonetheless, there are research gaps regarding the histopathological study of blood vessels under normal and hypercholesterolemic conditions. Future research into microencapsulated curcumin as a lipid-modulating agent is therefore required to be explored. This will involve evaluating cholesterol deposition in blood vessels using histopathological, immunohistochemical, and genomic indicators, and especially correlating the findings with human study.

## Data availability statement

The original contributions presented in this study are included in the article/supplementary material, further inquiries can be directed to the corresponding author/s.

## Ethics statement

The animal study was reviewed and approved by the Directorate of Graduate Studies, UAF (No. DGS/474-79) after ensuring the standards for handling and care of laboratory animals from the Departmental Bioethics Committee.

## Author contributions

HA conceptualized, designed and conducted the research experiments. MB co-conceptualized and provided technical assistance in designing the research. I-U-H, HA, and MN analyzed the results, drafted the manuscript, and wrote the original draft. RA and MT technically modified the manuscript concerning data visualization, language and editing. AR, I-U-H, RA, and MT contributed to the review and editing. All authors approved the final version of this manuscript.
